# Validation of somatic cell score-associated SNPs from Holstein cattle in Sudanese Butana and Butana × Holstein crossbred cattle

**DOI:** 10.1007/s11250-022-03048-3

**Published:** 2022-01-12

**Authors:** Salma Elzaki, Paula Korkuc, Danny Arends, Monika Reissmann, Siham A. Rahmatalla, Gudrun A. Brockmann

**Affiliations:** 1 Albrecht Daniel Thaer-Institute for Agricultural and Horticultural Sciences, Humboldt-Universität Zu Berlin, Berlin, Germany; 2 Department of Genetics and Animal Breeding, Faculty of Animal Production, University of Khartoum, Khartoum, Sudan; 3 Department of Dairy Production, Faculty of Animal Production, University of Khartoum, Khartoum, Sudan

**Keywords:** Association analysis, *Bos indicus*, Genotyping, Mastitis, SNPs

## Abstract

The *Bos indicus* zebu cattle Butana is the most commonly used indigenous dairy cattle breed in Sudan. In the last years, high-yielding Holstein dairy cattle were introgressed into Butana cattle to improve their milk yield and simultaneously keep their good adaption to extreme environmental conditions. With the focus on the improvement of milk production, other problems arose such as an increased susceptibility to mastitis. Thus, genetic selection for mastitis resistance should be considered to maintain healthy and productive cows. In this study, we tested 10 single nucleotide polymorphisms (SNPs) which had been associated with somatic cell score (SCS) in Holstein cattle for association with SCS in 37 purebred Butana and 203 Butana × Holstein crossbred cattle from Sudan. Animals were genotyped by competitive allele-specific PCR assays and association analysis was performed using a linear mixed model. All 10 SNPs were segregating in the crossbred Butana × Holstein populations, but only 8 SNPs in Sudanese purebred Butana cattle. The SNP on chromosome 13 was suggestively associated with SCS in the Butana × Holstein crossbred population (rs109441194, 13:79,365,467, *P*_BF_ = 0.054) and the SNP on chromosome 19 was significantly associated with SCS in both populations (rs41257403, 19:50,027,458, Butana: *P*_BF_ = 0.003, Butana × Holstein: *P*_BF_ = 6.2 × 10^−16^). The minor allele of both SNPs showed an increase in SCS. Therefore, selection against the disadvantageous minor allele could be used for genetic improvement of mastitis resistance in the studied populations. However, investigations in a bigger population and across the whole genome are needed to identify additional genomic loci.

**Supplementary Information:**

The online version contains supplementary material available at 10.1007/s11250-022-03048-3.

## Introduction

Livestock plays a significant role in the production of food and is of great socio-economic and cultural value in various societies around the world. In Sudan, the *Bos indicus* zebu cattle Butana are well adapted to the hot climate, harsh environment with low feed availability, the lack of water, and tropical diseases and parasites. But most importantly, they have a higher milk yield than reported from most African breeds (Ageeb and Hillers 2021). Butana cattle have an average milk yield of 538.3 kg per lactation under field conditions (Musa et al. 2005; 2006), and 1,662.6 kg under improved feeding and management conditions in a research station (Musa et al. 2005; 2006). The age at first calving ranges from 1168 to 1588 days and the lactation length was reported from 190 to 268 days (Ageeb and Hillers 2021; Musa et al. 2005; 2006). In order to improve milk production and reproduction traits, Butana cattle have been crossed with high-yielding Holstein dairy cattle. The resulting Butana × Holstein crossbred cattle are as robust as Butana cattle but have higher milk yield compared to purebred Butana with the milk yield per lactation being on average 3988.2 kg (Elnazeir et al. 2019). The lactation length is on average 356 days, and the age at first calving 1040 days (Eshraga 2011). Thus, crossbred Butana × Holstein cattle are currently used as the main source of milk in urban regions, but they can also be found in rural areas.

One of the main problems of dairy production in Sudan and worldwide is the high incidence of mastitis, an inflammatory udder disease causing economic losses due to reduced milk production and quality, and increased treatment costs or culling (Bar et al. 2008; Mungube et al. 2005). In cattle breeding, the somatic cell count (SCC) in milk is used as an indirect marker to monitor mastitis infections. A SCC higher than 100,000 cells/ml corresponding to a somatic cell score (SCS) of 3 is suggestive for a subclinical mastitis infection (Peeler et al. 2000). Because of the low heritability of clinical mastitis and SCS, traditional methods of selection have limited success (Hinrichs et al. 2011). Thus, selection using genomic markers has potential to improve mastitis resistance (Meuwissen et al. 2001).

Many studies in different Holstein populations have been performed, which identified genomic loci associated with SCS or clinical mastitis on chromosomes 1, 5, 6, 8, 13, 16, 18, 19, and 20 (Abdel-Shafy et al. 2014, 2018; Viale et al. 2017; Jiang et al. 2019). In this study, we tested 10 single nucleotide polymorphisms (SNPs) that have been previously associated with SCS in German Holstein bulls and cows (Abdel-Shafy et al. 2014) for associations with SCS in Sudanese purebred Butana and Butana × Holstein crossbred cattle. Five of those SNPs were also found to be associated with clinical mastitis (Abdel-Shafy et al. 2018). If those SNPs would be significantly associated, they could also be used as genetic markers to improve resistance against mastitis in Sudanese purebred Butana and Butana × Holstein crossbred populations. Such markers could contribute to improve the productivity of these cattle which is necessary to meet the increasing demand for milk and dairy products in Sudan.

## Material and methods

### Animals and phenotypic data

Data was obtained from 37 unrelated purebred Butana cows from Atbara research station in North Sudan and from 203 Butana × Holstein crossbred cows with varying levels of exotic blood belonging to five farms in Khartoum North. The cows were in lactation 1 to 8 with lactations of 5 and above being set to 5 + for statistical analyses. In this study, the average lactation length of Butana cattle was 243 days and of Butana × Holstein crossbred cattle 309 days. The age at first calving of Butana cattle was on average 1896 days and of Butana × Holstein crossbred cattle 998 days. One Butana cow was removed because the age at first calving was 3000 days and thereby more than 50% higher than the average of the population.

Milk samples were taken on multiple dates in 2017 and 2018. The somatic cell count (SCC) of milk samples was determined using the microscopic slide method which is a technique for the estimation of the number of nucleated somatic cells in unprocessed milk (Brazis et al. 1968). SCC was transformed to somatic cell score (SCS = log2 (SCC/100,000 cells/ml) + 3) in order to obtain a distribution close to normal. The total dataset included 1,261 records for SCS with on average 5.25 measurements per cow. Average SCS was calculated by first taking the mean SCS for a cow across all sample dates, and then calculating the mean SCS by breed and farm. Outliers were defined as values outside the mean ± 3 standard deviations. After filtering, 1,222 SCS measurements were available for 34 purebred Butana cows from Atbara research station (134 test-day records) and for 201 Butana × Holstein crossbred cows from five farms (1,088 test-day records).

### DNA isolation and SNP genotyping

Blood samples of all cows were collected from the jugular vein in sterile tubes containing EDTA as anticoagulant. DNA was isolated using a salting out procedure (Miller et al. 1988). Genotyping of 10 SNPs that have been identified for SCS (Abdel-Shafy et al. 2014) was performed with allele-specific primers using competitive allele-specific PCR assays (KASP) (Kreuzer et al. 2013). Primer information for SNPs genotyped in this study is fully provided in Abdel-Shafy et al. (Abdel-Shafy et al. 2014). Genomic positions were referenced to the current *Bos taurus* ARS_UCD2.1 genome assembly (Rosen et al. 2018).

### Association analysis

Association analysis between SCS and genotypic data was performed with a linear mixed model using lmer function implemented in R language for statistical computing. Butana and Butana × Holstein crossbred cattle were examined separately. The model for testing the additive effect for each SNP in Butana cattle was:

1 $$\mathrm{Y }=\mathrm{ SY }+\mathrm{ DIM }+\mathrm{ GT}+ (1|\mathrm{animal}) +\mathrm{ e}$$

where Y is the SCS from multiple sample dates and SY represents the covariate for the year of sampling followed by the covariate for days in milk DIM and the SNP genotype GT. The animal was included as a random effect (1|animal) to compensate for repeated measurements; e is the residual error.

The model for testing the additive effect for each SNP in Butana × Holstein crossbred cattle was

2 $$\mathrm{Y }=\mathrm{ FA }+\mathrm{ SY }+\mathrm{ CS }+\mathrm{ FC }+\mathrm{ DIM }+\mathrm{ GT }+ (1|\mathrm{animal}) +\mathrm{ e}$$

where the same formula was used as in Eq. 1 plus the covariates for farm FA, calving season CS (dry season from November to April; wet season from May to October), and age at first calving in days FC.

The covariates year of sampling, days in milk, calving season, age at first calving, and farm were only included as fixed effects into the model, if the covariate significantly contributed to the model (Supplementary Table 1). This was tested by performing an ANOVA test between the null model (Y = (1|animal)) and the null model extended with one of the covariates (Y = covariate + (1|animal)). Bonferroni correction was used to adjust *P*-values for the 10 tested SNPs. *P*-values after Bonferroni correction (*P*_BF_) were considered suggestive if *P*_BF_ < 0.1 and significant if *P*_BF_ < 0.05. SNP effect plots were done using R package ggplot2 (version 3.3.2) and *P*-values between genotype groups were estimated using pairwise *t*-tests and displayed using R package ggpubr (version 0.4.0). For the SNP effect plots, SCSs were corrected for each breed separately using significant covariates from Eq. 1 and Eq. 2 and then averaged across the animal identification numbers.

## Results

**Fig. 1:**
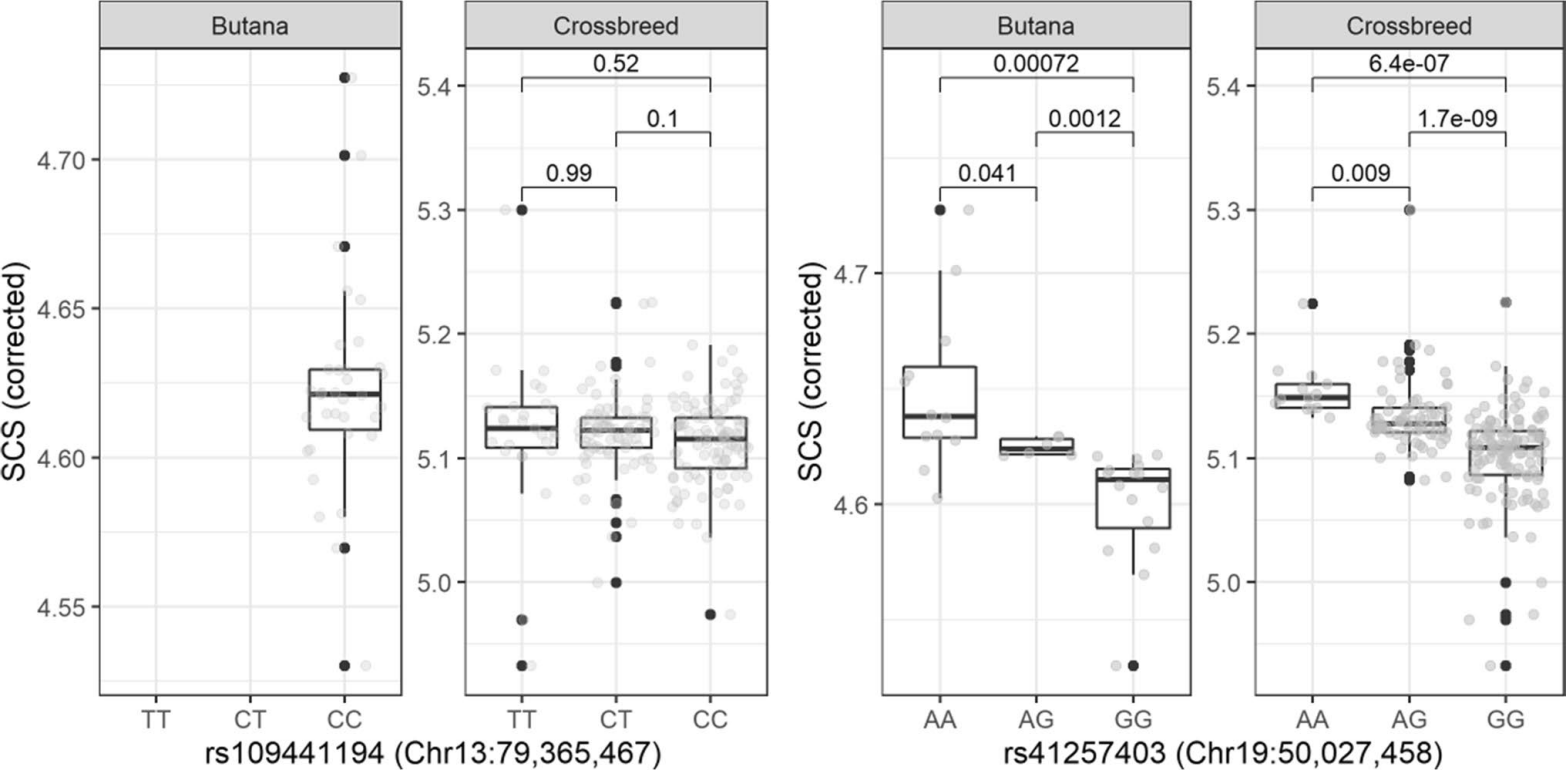
SNP effect plots for the significantly associated SNPs on chromosomes 13 (rs109441194) and 19 (rs41257403) for Butana and Butana × Holstein cows. The boxplots show the corrected averaged SCS for each SNP genotype group. Individual animals are visualized by jittered points. *P*-values between genotype groups from two-sided *t*-tests are displayed

SCS was with 4.6 lower in Butana cattle compared to on average 5.1 in Butana × Holstein crossbred cattle, whereas the mean SCS per farm ranged from 4.8 to 5.3 (Supplementary Table 2). We observed that all 10 investigated SNPs were segregating in Butana × Holstein crossbred cattle, but only eight in Butana cattle (Table 1). Two out of the 10 previously reported SNPs were associated with SCS in Butana × Holstein crossbreed cattle (Fig. 1). One suggestively associated SNP was located on chromosome 13 (rs109441194, 13:79,365,467, *P*_BF_ = 0.054). This SNP had a minor allele frequency (MAF) of 0.33 and its minor allele T accounted for an increase in SCS of 0.254. The other SNP located on chromosome 19 was significantly associated with SCS (rs41257403, 19:50,027,458, *P*_BF_ = 6.2 × 10^−16^). The minor allele A of the chromosome 19 SNP had a frequency of 0.24 and increased SCS by 0.726.

**Table 1:** Association results for SCS in Butana and Butana × Holstein crossbred cattle. Each tested SNP is listed with its SNP ID, chromosome number (Chr), genomic positions in bp with regard to the *Bos taurus* ARS_UCD1.2 genome assembly, the minor allele (A1), the major allele (A2), the minor allele frequency (*F*_A1_), the *β*-estimate per minor allele change (*β*_A1_), the standard error of the *β*-estimate (SE (*β*_A1_)), and the Bonferroni-corrected *P*-value of the association test (*P*_BF_). *P*_BF_ < 0.1 are highlighted in bold. Two SNPs were not segregating in Butana. Thus, no association analysis could be calculated with them

SNP ID	Chr	Position	A1	A2	Butana				Butana × Holstein crossbred			
					***F***_**A1**_	***β***_**A1**_	**SE (*β***_**A1**_**)**	***P***_**BF**_	***F***_**A1**_	***β***_**A1**_	**SE (*β***_**A1**_**)**	***P***_**BF**_
rs41257360	5	97,477,421	G	A	0.000	-	-	-	0.216	− 0.159	0.112	1
rs41588957	6	83,803,915	C	T	0.190	0.087	0.313	1	0.240	− 0.091	0.104	1
rs110707460	6	86,337,334	G	A	0.300	0.139	0.314	1	0.206	0.133	0.102	1
rs109934030	13	77,914,930	T	C	0.014	− 0.103	0.541	1	0.100	0.167	0.158	1
rs41634110	13	79,002,832	G	A	0.041	− 0.342	0.635	1	0.181	0.220	0.121	1
rs109441194	13	79,365,467	T	C	0.000	-	-	-	0.326	0.254	0.090	**0.054**
rs29020544	18	43,157,279	T	G	0.140	− 0.077	0.373	1	0.382	0.009	0.102	1
rs41257403	19	50,027,458	A	G	0.460	0.849	0.149	**0.003**	0.243	0.726	0.087	**6.2 × 10**^−**16**^
rs41636878	19	51,815,015	T	C	0.200	− 0.497	0.335	1	0.228	0.007	0.116	1
rs41629005	X	30,341,984	T	C	0.041	0.388	0.759	1	0.431	− 0.018	0.093	1

In the investigated Butana cattle, the SNP on chromosome 13 that was suggestive in the crossbred cattle was not segregating, but the SNP on chromosome 19 was also significantly associated with SCS in Butana (rs41257403, 19:50,027,458, *P*_BF_ = 0.003). The minor allele was the same as in the crossbred population and had the same direction and a similar magnitude of effect. The frequency of the unfavourable minor allele A of the chromosome 19 SNP in the purebred Butana population was 0.46, increasing SCS by 0.849.

## Discussion

In the current study, we confirmed suggestive and significant effects on SCS of the two SNPs rs109441194 and rs41257403 on chromosomes 13 and 19, respectively, in Butana × Holstein crossbreed cattle. In Butana cattle, the SNP on chromosome 19 was also significantly associated with SCS, while the SNP on chromosome 13 was monomorphic. Therefore, the chromosome 13 region could not be tested for potential association of the linked chromosomal region with SCS in Butana. These two confirmed SNPs were not only associated with SCS but also directly with clinical mastitis in Holstein cows (Abdel-Shafy et al. 2018) which makes the findings more important. In the regions of the two confirmed SNPs are the same two positional candidate genes as reported previously (Abdel-Shafy et al. 2014, 2018): NFATC2 (cytoplasmic nuclear factor of activated T-cells 2) and FOXK2 (forkhead box protein K2), which are both linked directly or indirectly to immune response of T-cells.

The MAFs of all tested SNP ranged from 0.00 to 0.46 in Butana and 0.10 to 0.43 in Butana × Holstein crossbred cattle (Table 1). Across all 10 SNPs, the difference of allele frequency was bigger between Holstein (Abdel-Shafy et al. 2014) and Butana cattle (average difference of MAFs = 0.17) than between Holstein and Butana × Holstein crossbred cattle (average difference of MAFs = 0.10). For the two confirmed SNPs (rs109441194, rs41257403), the MAFs were similar in Holstein and in Butana × Holstein crossbred cattle (difference of MAFs = 0.04). With respect to the SNP on chromosome 19, the MAF was much higher in Butana cattle, where the SNP was fixed (difference of MAF = 0.26).

The directions of effects of the two confirmed SNPs were the same in both investigated breeds. In all cases, the minor allele was disadvantageous increasing SCS. Therefore, selection against the minor allele could reduce the somatic cell numeric in the milk in the population and thereby hopefully improve resistance against clinical mastitis. Comparing to previous results in Holstein bulls and cows (Abdel-Shafy et al. 2014), the direction of effects of the associated SNP on chromosome 13 was consistent meaning that the same region is linked to SCS or immune response in Butana × Holstein cattle as in Holstein Friesian cattle. In contrast, the associated SNP on chromosome 19 showed an opposite direction of effects compared to previous results in Holstein cattle (Abdel-Shafy et al. 2014) which could be caused by the weak linkage between the investigated SNP and the causative mutation.

In general, SCC and thus SCS were high in the investigated Butana (average SCS = 4.6) and Butana × Holstein crossbred cattle (average SCS = 5.1). In contrast, SCS in different Holstein herds ranges between 2.91 and 4.62 (da Silva et al. 2018; Toffanin et al. 2015; Costa et al. 2019). The SCS of milk that is allowed to be consumed varies in different countries, e.g., in the USA, Canada, and Germany, and the SCS threshold is set to 5.9, 5.3, and 5.0, respectively (Olechnowicz and Jaśkowski 2012; Schwarz et al. 2011). The high SCS in Sudanese cattle may indicate that Sudanese farms have high rates of subclinical mastitis or even clinical mastitis. First and foremost, better farm management, higher hygienic requirements, and proper nutrition of cattle are necessary on Sudanese farms in order to reduce mastitis cases. However, a better understanding of the genetic background of mastitis resistance, more accurate phenotyping, and genetic selection using mastitis markers could improve resistance against mastitis.

The biggest pitfall of this study is the low sample size, especially of Butana cattle, where alleles might be missed due to low MAFs. Since the sample size of Butana × Holstein crossbred cattle was larger, higher significance was obtained for these cattle. Nonetheless, we could confirm two SNPs to be associated with SCS in Butana × Holstein crossbred cattle and one in Butana cattle. These are SNPs which could be used for genetic improvement of mastitis resistance in the respective breeds. In order to validate the other SNPs, higher number of animals would be needed.

This is the first association study for SCS in Sudanese Butana and Butana × Holstein crossbred cattle. The genetic improvement of mastitis resistance and selection for lower SCS is consistent with the goal of maximizing genetic improvement for milk production and total economic merit and should be included in breeding programs. We confirmed the association of two SNPs in Butana × Holstein crossbred cattle, whereof one was also significant in purebred Butana cattle. These SNPs can be used for genomic selection to reduce SCS in milk and thus to improve mastitis resistance and further milk quality. Follow-up associations in bigger populations using SNP-chip or whole-genome sequencing data would allow for the identification of new loci associated with SCS.

## Supplementary Information

Below is the link to the electronic supplementary material.

Supplementary file1 (XLSX 148 KB)

## Data Availability

All genotypes and phenotypes analysed are included in Supplementary Table 3.
